# Clinical Impact of Renal Dysfunction in Patients with Severe Tricuspid Regurgitation and Chronic Heart Failure

**DOI:** 10.31083/RCM26080

**Published:** 2025-03-05

**Authors:** Beniamino Rosario Pagliaro, Pier Pasquale Leone, Alessandro Villaschi, Francesca Pugno Vanoni, Matteo Biroli, Ferdinando Loiacono, Marta Pellegrino, Giuseppe Pinto, Marta Maccallini, Matteo Pagnesi, Giuliana Cimino, Laura Lupi, Damiano Regazzoli Lancini, Renato Maria Bragato, Giulio Stefanini, Bernhard Reimers, Daniela Pini, Marco Metra, Gianluigi Condorelli, Marianna Adamo, Antonio Mangieri, Antonio Colombo

**Affiliations:** ^1^IRCCS Humanitas Research Hospital, 20089 Rozzano, Milan, Italy; ^2^Department of Biomedical Sciences, Humanitas University, 20072 Pieve Emanuele, Milan, Italy; ^3^Department of Clinical and Molecular Medicine, Sapienza University, 00185 Rome, Italy; ^4^Cardiology and Cardiac Catheterization Laboratory, ASST Spedali Civili di Brescia and Department of Medical and Surgical Specialties, University of Brescia, 25123 Brescia, Italy

**Keywords:** tricuspid regurgitation, chronic heart failure, chronic kidney disease, right ventricular dysfunction

## Abstract

**Background:**

Renal dysfunction (RD) is common in patients with heart failure (HF), however its impact on clinical outcomes in patients with tricuspid regurgitation (TR) and HF is still debated; therefore, we aimed to assess the impact of RD on clinical outcomes in this population.

**Methods:**

All patients with HF and a prevalent or incident diagnosis of TR presenting at two centers between January 2020 and July 2021 were enrolled, in both acute (in-hospitalized patients) and chronic settings (outpatient). Patients were stratified according to the degree of RD (Group 1 <30 mL/min (n = 70), Group 2 30–59 mL/min (n = 123) and Group 3 ≥60 mL/min (n = 56).

**Results:**

Out of 249 patients, those with severe RD had lower left ventricular ejection fraction (41.8 ± 13.1% vs. 45.7 ± 14.2% vs. 48.6 ± 13.1%, *p* = 0.020) and tricuspid annular plane systolic excursion (16.6 ± 3.7 mm vs. 17.6 ± 4.0 mm vs. 20.0 ± 4.4 mm, *p* < 0.001) while brain natriuretic peptides levels were higher (979 ± 1514 pg/mL vs. 490 ± 332 pg/mL vs. 458 ± 543 pg/mL, *p* = 0.049) than in the other subgroups. After a median follow-up of 279 (interquartile range, IQR 195–481) days, all-cause mortality was higher in patients with severe RD (37.7% vs. 23.3% vs. 13.7%, *p* = 0.012). HF hospitalizations (32.7% vs. 31.2% vs. 30.6%, *p* = 0.970) and the composite of all-cause mortality or HF hospitalization (54.1% vs. 47.9% vs. 42.0%, *p* = 0.444) did not differ between subgroups.

**Conclusions:**

Severe RD is highly present in patients with HF and TR and is associated with increased incidence of all-cause mortality.

## 1. Introduction

Renal dysfunction (RD) is a common comorbidity in patients with heart failure (HF), ranging from 30% to 50% of patients, and is linked to poorer outcomes across all HF phenotypes [1]. The heart and kidneys share a close relationship, where dysfunction in one organ can lead to the deterioration of the other through various mechanisms, including inflammation, oxidative stress, disrupted fluid balance, and diuretic resistance [2, 3]. As an example, in chronic HF, reduced cardiac output and increased filling pressures lead to diminished organ perfusion. This reduction in perfusion can activate compensatory mechanisms within the kidneys that are intended to preserve circulatory volume and blood pressure, but ultimately worsen congestion. The kidneys respond by increasing the reabsorption of water and sodium through the activation of renin-angiotensin-aldosterone system (RAAS) and the release of antidiuretic hormone (ADH). This triggers the kidneys to retain water and sodium as a compensatory response, resulting in subclinical or clinical congestion, which further exacerbates RD [4]. Recent findings highlight a significant link between RD and venous congestion, as the latter may contribute to the onset of congestive kidney failure by increasing renal afterload and interstitial pressure within the kidneys [5, 6]. The presence of venous congestion, rather than reduced forward flow, is now considered a key determinant of worsening renal function in HF patients, underscoring the importance of managing fluid balance to preserve kidney function [4]. Right ventricular (RV) dysfunction and functional tricuspid regurgitation (FTR) can exacerbate venous congestion, thereby affecting renal function. For instance, a tricuspid annular plane systolic excursion (TAPSE) ≤14 mm has been closely linked to a higher prevalence of RD and increased mortality risk in patients with chronic systolic HF [7]. In patients with reduced TAPSE, the interplay between impaired RV function and kidney congestion becomes particularly important, as both contribute to a vicious cycle of worsening heart and kidney function [5]. Additionally, patients with HF and moderate-to-severe FTR often exhibit more pronounced signs of congestion, leading to the hypothesis that FTR may also play a role in RD [8]. However, despite the recent interest in tricuspid regurgitation (TR) and HF, literature data on the clinical impact of RD in patients with severe TR and HF are scarce. While many studies [2, 3, 4, 5, 6, 7] have explored the consequences of RD in the broader HF population, less is known about how RD specifically impacts outcomes in patients with TR, particularly those with severe regurgitation. This gap in the literature is important, as the combination of TR, venous congestion, and RD may represent a unique phenotype of HF with particularly poor prognosis [9]. Therefore, we aimed to assess the incidence of mortality and hospitalizations for HF in patients with TR and HF according to the degree of RD.

Understanding the role of RD in these patients may have significant implications for risk stratification and therapeutic approaches, potentially guiding more personalized treatment strategies to improve outcomes in this high-risk population.

## 2. Materials and Methods

All patients with HF and a prevalent or incident diagnosis of TR presenting at two centers between January 2020 and July 2021 were enrolled, in both acute (in-hospitalized patients) and chronic settings (outpatient). All patients received echocardiographic assessments at the echocardiography laboratories of the participating institutions. Inclusion criteria were as follows: patients aged 18 years or over with established clinical diagnosis of chronic HF with reduced or preserved ejection fraction as recommended by most recent European Society of Cardiology (ESC) Acute and Chronic HF guidelines [10]; severe TR (organic or functional), diagnosed by two-dimensional (2D) transthoracic echocardiography according to European Association of Cardiovascular Imaging (EACVI) recommendations for the echocardiographic assessment of native valvular regurgitation [11]. The exclusion criteria included clinical or echocardiographic signs of pericardial, congenital, or infiltrative heart disease, recent acute myocardial infarction, and a suboptimal echocardiographic window that hindered the complete quantification of TR or accurate anatomical evaluation. Data regarding the echocardiographic evaluation are described in **Supplementary Material**: briefly, severity of mitral and TR were assessed as per current guidelines [11], RV dimensions were measured using the end-diastolic mid-ventricular diameter, and RV systolic function was assessed with TAPSE, where a value below 17 mm indicated RV systolic dysfunction. Data collected at the time of the initial visit or echocardiogram included New York Heart Association (NYHA) class, valvular procedures, laboratory biomarkers (such as serum creatinine, brain natriuretic peptide (BNP) or N-terminal pro brain natriuretic peptide, (NT-proBNP)), medical treatments, and comorbidities including hypertension, diabetes mellitus, atrial fibrillation, and chronic obstructive pulmonary disease. The estimated glomerular filtration rate (eGFR) was calculated using the Chronic Kidney Disease Epidemiology Collaboration (CKD-EPI) equation. For renal function and natriuretic peptide levels, values obtained within two months of follow-up under stable clinical conditions were used as index values.

Patients were divided according to the eGFR into severe RD (Group 1, eGFR <30 mL/min/1.73 m^2^), moderate RD (Group 2, eGFR 30–59 mL/min/1.73 m^2^) and mild RD or preserved renal function (Group 3, eGFR ≥60 mL/min/1.73 m^2^), according to Kidney Disease Improving Global Outcomes (KDIGO) clinical practice guidelines for the evaluation and management of chronic kidney disease (CKD) [12].

During follow-up, patients were re-evaluated at regular intervals every 3–6 months through follow-up visits. If a visit was missed, the patient’s vital status was confirmed via telephone contact performed by specialized nurses who routinely telephone follow up with patients who miss scheduled visits. For these cases, the nurses conduct a brief telephone assessment to evaluate the patient’s vital status and health condition. This process ensured data completeness without altering the retrospective design of the study, as it reflects standard clinical practice in our HF units. All data from these telephone contacts were collected retrospectively from medical records, aligning with the study’s retrospective design.

The study was carried out in accordance with the guidelines of the Declaration of Helsinki and approved by the Ethics Committee of Humanitas Research Hospital (Protocol number is 85/24). Due to the study design, written informed consent was not required.

### 2.1 Study Endpoints

The primary endpoint was incidence of all-cause mortality. Secondary endpoints were incidence of HF hospitalization and incidence of the composite endpoint including all-cause mortality or HF hospitalizations.

### 2.2 Statistical Considerations

Continuous variables are reported as median (interquartile range, IQR) and were compared using Student’s *t*-test or the Mann-Whitney U based on the normality of data distribution, verified using the Kolmogorov-Smirnov goodness-of-fit test. Categorical variables are reported as number (percentage) and were compared using the χ^2^ test without Yates’ correction for continuity or the Fisher’s exact test as appropriate. Time-to-event analysis was performed according to Kaplan-Meier, and groups were compared with log-rank test. Hazard ratio (HR) and 95% confidence intervals (CI) for the outcomes were calculated with univariable and multivariable Cox regression. Variables included in multivariable regression models were selected based on clinical relevance. Clinical follow-up was censored at the date of death or last contact. Two-sided *p* values < 0.05 were considered statistically significant. Statistical analyses were performed using Stata (V.16.0, StataCorp LLC, College Station, TX, USA).

## 3. Results

The overall population (N = 249) was divided into 3 groups according to the eGFR: group (1) patients with severe RD (eGFR <30 mL/min/1.73 m^2^; N = 70, 26%); group (2) patients with moderate RD (eGFR 30–59 mL/min/1.73 m^2^; N = 123, 46%); group (3) patients with mild RD or preserved renal function (eGFR ≥60 mL/min/1.73 m^2^; N = 56, 21%). As reported in Table 1, mean age of overall population was 79.3 ± 9.0 years old with 51.8% of male sex. Age was comparable between groups (80.3 ± 7.8 years vs. 79.8 ± 8.5 vs. 77.0 ± 10.9, *p* = 0.082), as was the proportion of male patients (50% vs. 55.3% vs. 46.4%, *p* = 0.513). The body mass index (BMI) was significantly higher in the Group 1 patients (26.3 ± 5.5 vs. 24.6 ± 4.4 vs. 26.1 ± 4.6, *p* = 0.044). The main cardiovascular risk factors as diabetes mellitus, arterial hypertension and hypercholesterolemia were equally distributed in all 3 groups of patients. Also, the presence of chronic obstructive pulmonary disease (COPD) did not differ into 3 groups (18.1% vs. 15.7% vs. 11.5%, *p* = 0.571). About a quarter of the overall population had history of myocardial infarction with similar distribution into 3 groups (32.9% vs. 24.3% vs. 17.9%, *p* = 0.149). The history of atrial fibrillation or atrial flutter was significantly more represented in the Group 1 patients in comparison with other two groups (88.9 vs. 80.6 vs. 62.3, *p* ≤ 0.001). When compared with patients with moderate or mild/no RD, the BNP and NT-proBNP values were higher in patients with severe RD (BNP: 979 ± 1514 pg/mL vs. 490 ± 332 pg/mL vs. 458 ± 543 pg/mL, *p* = 0.049; NT-proBNP: 12,465 ± 14,743 vs. 7070 ± 8274 vs. 8095 ± 9751, *p* = 0.002). Similarly, the Society of Thoracic Surgery (STS) Predicted Risk of Mortality was higher in patients with severe RD (11.4 ± 6.5% vs. 7.2 ± 4.7% vs. 5.7 ± 3.9%, *p* ≤ 0.001). The overall population was highly symptomatic for HF with 40.9% of NYHA class III–IV and NYHA class did not differ significantly between patient groups (45.8% vs. 42% vs. 32%, *p* = 0.547). The presence of implantable cardioverter-defibrillators (ICD) or cardiac resynchronization therapy did not differ into three groups of patients (29.3% vs. 35.5% vs. 13%, *p* = 0.168).

**Table 1.** **Baseline clinical characteristics**.

Variable	Overall (N = 249)	Group 1	Group 2	Group 3	*p*-value
		eGFR <30 mL/min/1.73 m^2^	eGFR 30–59 mL/min/1.73 m^2^	eGFR ≥60 mL/min/1.73 m^2^	
		(N = 70, 26%)	(N = 123, 46%)	(N = 56, 21%)	
Age (years)	79.3 ± 9.0	80.3 ± 7.8	79.8 ± 8.5	77.0 ± 10.9	*p* = 0.082
Male Sex (%)	138 (51.8)	35 (50)	68 (55.3)	26 (46.4)	*p* = 0.513
BMI (kg/m^2^)	**25.4 ± 4.8**	**26.3 ± 5.5**	**24.6 ± 4.4**	**26.1 ± 4.6**	***p* = 0.044**
Diabetes Mellitus (%)	84 (31.5)	30 (41.7)	38 (28.3)	16 (26.2)	*p* = 0.088
Hypertension (%)	192 (71.9)	56 (77.8)	96 (71.6)	40 (65.6)	*p* = 0.295
Hypercholesterolemia (%)	137 (51.3)	38 (52.8)	65 (48.5)	34 (55.7)	*p* = 0.962
Cancer in the Previous Year (%)	44 (16.5)	13 (18.0)	24 (17.9)	7 (11.5)	*p* = 1.439
Previous AMI (%)	68 (25.5)	23 (31.9)	34 (25.4)	11 (18.0)	*p* = 0.149
Previous PCI (%)	57 (21.3)	18 (25.0)	29 (21.6)	10 (16.3)	*p* = 1.470
Previous CABG (%)	34 (12.7)	13 (18.1)	17 (12.7)	4 (6.5)	*p* = 0.140
Previous Stroke/TIA (%)	44 (16.5)	13 (18.1)	22 (16.4)	9 (14.8)	*p* = 0.262
COPD (%)	41 (15.3)	13 (18.1)	21 (15.7)	7 (11.5)	*p* = 0.571
History of AF/AFL (%)	**210 (78.6)**	**64 (88.9)**	**108 (80.6)**	**38 (62.3)**	***p* < 0.001**
BNP (pg/mL)	**618 ± 885**	**978 ± 1514**	**490 ± 332**	**457 ± 543**	***p* = 0.049**
NT-proBNP (pg/mL)	**9007 ± 11,163**	**12,465 ± 14,743**	**7070 ± 8274**	**8095 ± 9751**	***p* = 0.002**
NYHA Class III–IV (%)	108 (40.9)	33 (45.8)	55 (42)	20 (32)	*p* = 0.547
STS (%)	**7.7 ± 5.4**	**11.5 ± 6.4**	**6.9 ± 4.7**	**6.1 ± 4.1**	***p* < 0.001**
ICD/CRT-D/CRT-P (%)	81 (30)	21 (29.3)	47 (35.5)	13 (21)	*p* = 0.168

eGFR, estimated glomerular filtration rate; BMI, body mass index; AMI, acute myocardial infarction; PCI, percutaneous coronary intervention; CABG, coronary-artery by-pass graft; TIA, transient ischemic attack; COPD, chronic obstructive pulmonary disease; AF, atrial fibrillation; AFL, atrial flutter; BNP, brain natriuretic peptide; NT-proBNP, N-terminal pro brain natriuretic peptide; NYHA, New York Heart Association; STS, Society of Thoracic Surgery; ICD, implantable cardioverter defibrillator; CRT, cardiac resynchronization therapy; CRT-D, cardiac resynchronization therapy defibrillator; CRT-P, cardiac resynchronization therapy pacemaker. Statistically significant variables have been highlighted in bold.

### 3.1 Echocardiographic Data

As reported in Table 2, both left ventricular ejection fraction (41.8 ± 13.1% vs. 45.7 ± 14.2% vs. 48.6 ± 13.1%, *p* = 0.020) and tricuspid annular plane systolic excursion (16.6 ± 3.7 mm vs. 17.6 ± 4.0 mm vs. 20 ± 4.4 mm, *p* < 0.001) were lower in patients with severe RD (Group 1). Moreover, the right atrial volume was significantly higher in Group 2 patients when compared with other groups (88.3 ± 32 mL vs. 111 ± 32 mL vs. 86.6 ± 41 mL, *p* = 0.039). All the other parameters measured by echocardiographic examination did not significantly differ between the 3 groups of patients.

**Table 2.** **Echocardiographic parameters**.

Variable		Overall (N = 249)	Group 1	Group 2	Group 3	*p*-value
			eGFR <30 mL/min/1.73 m^2^	eGFR 30–59 mL/min/1.73 m^2^	eGFR ≥60 mL/min/1.73 m^2^	
			(N = 70, 26%)	(N = 123, 46%)	(N = 56, 21%)	
LA Volume (mL)		120 ± 57	111 ± 41	129 ± 61	111 ± 60	*p* = 0.098
LVEDD (mm)		54.1 ± 10	54 ± 9	54.8 ± 10	53 ± 10	*p* = 0.430
LVEDV (mL)		120 ± 57	116 ± 57	126.5 ± 61	110.6 ± 48	*p* = 0.229
LVEDVi (mL/mq)		59.6 ± 23	60.3 ± 29	60.1 ± 22	58 ± 19	*p* = 0.083
LVEF (%)		**44.9 ± 14**	**41.7 ± 13**	**45.0 ± 14**	**48.5 ± 13**	***p* = 0.021**
Severe MR (3–4+/4+) (%)		128 (48)	32 (44)	67 (49)	29 (48)	*p* = 0.773
Mechanism of TR						*p* = 0.599
	Primary (%)	19 (7.3)	5 (7.1)	12 (9.2)	2 (3.3)	
	Secondary (%)	213 (81.9)	59 (84.2)	104 (80.0)	50 (83.3)	
	CIED-Related (%)	28 (10.8)	6 (8.6)	14 (10.8)	8 (13.3)	
RVEDD (mm)		44.7 ± 8	45.1 ± 8	45.2 ± 9	43.3 ± 6	*p* = 0.477
RVEDV (mL)		45.6 ± 20	48.2 ± 10	46.5 ± 11	44.7 ± 9	*p* = 0.587
TAPSE (mm)		**17.8 ± 4**	**16.7 ± 4**	**17.6 ± 4**	**19.8 ± 4**	***p* < 0.001**
RVEF (%)		54.0 ± 7	56.7 ± 10	52.0 ± 10	52.5 ± 5	*p* = 0.741
RA Volume (mL)		**99.1 ± 54**	**88.3 ± 32**	**111 ± 32**	**86.6 ± 41**	***p* = 0.039**
E/e’		13.7 ± 6	14.1 ± 8	14.5 ± 6	11.2 ± 4	*p* = 0.063
sPAP (mmHg)		51.4 ± 14	54.4 ± 17	51.2 ± 13	48.3 ± 12	*p* = 0.061

eGFR, estimated glomerular filtration rate; LA, left atrial; LVEDD, left ventricle end-diastolic diameter; LVEDV, left ventricle end-diastolic volume; LVEDVi, indexed left ventricle end-diastolic volume; LVEF, left ventricle ejection fraction; MR, mitral regurgitation; TR, tricuspid regurgitation; CIED, Cardiovascular Implantable Electronic Device; RVEDD, right ventricle end-diastolic diameter; RVEDV, right ventricle end-diastolic volume; TAPSE, tricuspid annular plane systolic excursion; RVEF, right ventricle ejection fraction; RA, right atrium; sPAP, systolic pulmonary arterial pressure. Statistically significant variables have been highlighted in bold.

### 3.2 Medical Therapy

As reported in Table 3, the percentage of RAAS inhibitors is very low in the overall population, especially in patients with severe RD. Utilization of Angiotensin-converting enzyme (ACE) inhibitors is significantly lower in Group 1 when compared with other two groups of patients (11.1% vs. 27.6% vs. 29.5%, *p* = 0.014). No significant difference was observed for other RAAS inhibitors drugs. A statistic borderline difference was observed in the 3 groups in relation to mineralocorticoid receptor antagonists (MRAs) administration (48.6% vs. 65.7% vs. 59%, *p* = 0.059). On the other hand, an important difference was reported about the average dosage of loop diuretics, which were significantly higher in patients with severe RD (215.8 ± 33 mg vs. 149.1 ± 17 mg vs. 94.4 ± 13 mg, *p* = 0.013).

**Table 3.** **Medical therapies**.

Variable (%)	Overall (N = 249)	Group 1	Group 2	Group 3	*p*-value
		eGFR <30 mL/min/1.73 m^2^	eGFR 30–59 mL/min/1.73 m^2^	eGFR ≥60 mL/min/1.73 m^2^	
		(N = 70, 26%)	(N = 123, 46%)	(N = 56, 21%)	
ACEi	**23.6**	**11.1**	**27.6**	**29.5**	***p* = 0.014**
ARBs	12	12.5	12.7	9.8	*p* = 0.840
ARNI	7.9	2.8	9.8	9.8	*p* = 0.172
Beta-Blockers	81.3	84.7	80.6	78.7	*p* = 0.647
Loop Diuretics	92.8	91.7	96.2	86.9	*p* = 0.141
Furosemide Equivalent (mg)	**153.6 ± 22**	**215.8 ± 33**	**149.1 ± 17**	**94.4 ± 13**	***p* = 0.013**
Thiazide Diuretics	6.8	6.9	8.7	2.2	*p* = 0.350
MRAs	59.5	48.6	65.7	59.0	*p* = 0.059
Ivabradine	1.5	0.0	2.2	1.6	*p* = 0.446
Digitalis	4.6	2.9	5.1	5.8	*p* = 0.723
SGLT2i	3	1.4	3.0	4.9	*p* = 0.493
Amiodarone	11.6	9.7	14.2	8.2	*p* = 0.406
Statins	41.1	43.7	41.3	37.7	*p* = 0.784

eGFR, estimated glomerular filtration rate; ACEi, angiotensin-converting enzyme inhibitors; ARBs, angiotensin receptor blockers; ARNI, angiotensin receptor neprilysin inhibitor; MRAs, mineralocorticoid receptor antagonists; SGLT2i, sodium-glucose co-transporter-2 inhibitors. Statistically significant variables have been highlighted in bold.

### 3.3 Clinical Outcomes

**Fig. 1.**
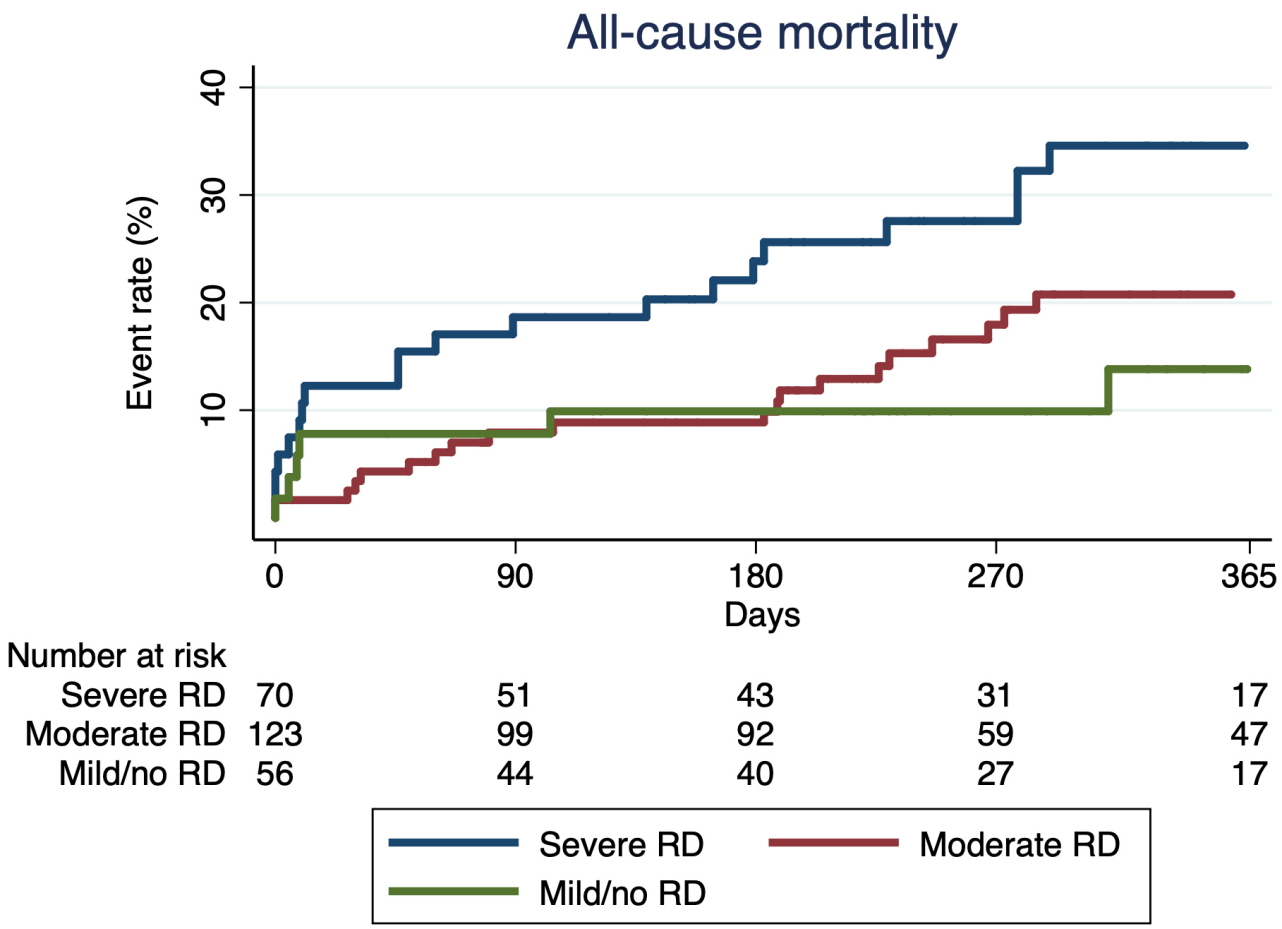
**All-cause mortality**. Kaplan – Meier curves for survival in patients with severe RD (eGFR <30 mL/min/1.73 m^2^) vs. those with moderate RD (eGFR 30–59 mL/min/1.73 m^2^) vs. those with mild RD or preserved renal function (eGFR ≥60 mL/min/1.73 m^2^). RD, renal dysfunction; eGFR, estimated glomerular filtration rate.

**Fig. 2.**
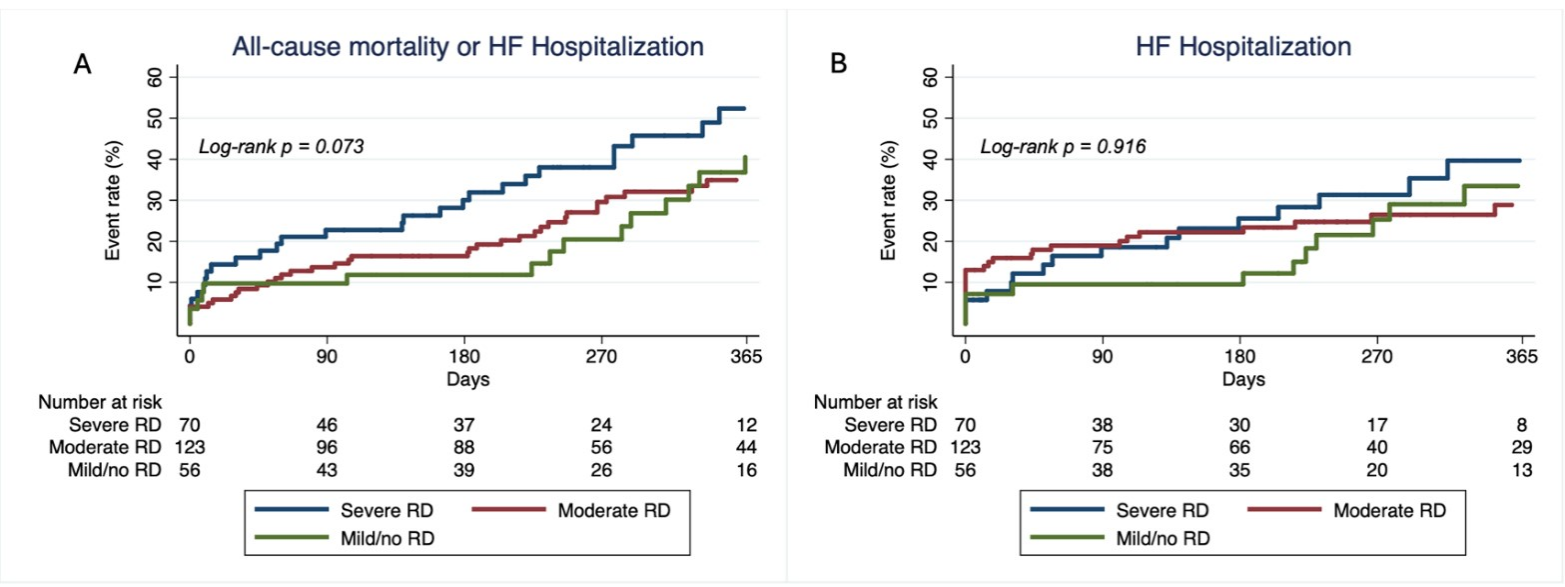
**All-cause mortality and HF hospitalization – HF hospitalization alone**. (A) Kaplan – Meier curves for the composite endpoint in patients with severe RD (eGFR <30 mL/min/1.73 m^2^) vs. those with moderate RD (eGFR 30–59 mL/min/1.73 m^2^) vs. those with mild RD or preserved renal function (eGFR ≥60 mL/min/1.73 m^2^). Log-rank *p* = 0.073. (B) Kaplan – Meier curves for first HF hospitalization in patients with severe RD (eGFR <30 mL/min/1.73 m^2^) vs. those with moderate RD (eGFR 30–59 mL/min/1.73 m^2^) vs. those with mild RD or preserved renal function (eGFR ≥60 mL/min/1.73 m^2^). HF, heart failure; eGFR, estimated glomerular filtration rate; RD, renal dysfunction. Log-rank *p* = 0.916.

Median follow-up was 279 (interquartile range 195–481) days. When compared to patients with moderate or mild/no RD, incidence of all-cause mortality was higher in patients with severe RD (37.7% vs. 23.3% vs. 13.7% for severe, moderate and mild/no RD, respectively, *p* = 0.012, Table 4). The all-cause mortality as estimated by Kaplan–Meier analysis, was significantly poorer in patients with severe RD at 1 year, compared to patients with moderate or mild/no RD (log-rank *p* = 0.014) (Fig. 1). Incidence of HF hospitalization did not differ between cohorts (32.7% vs. 31.2% vs. 30.6%, *p* = 0.970). Similarly, incidence of the composite outcome of all-cause mortality or HF hospitalization was similar when comparing groups (54.1% vs. 47.9% vs. 42.0%, *p* = 0.444). Using Kaplan-Meier analysis the composite endpoint of all-cause mortality or HF hospitalization tended to be higher in patients with worse RD (log-rank *p* = 0.073) while no difference was found for HF hospitalization alone (log-rank *p* = 0.916) (Fig. 2A,B).

**Table 4.** **Clinical outcomes**.

Variable (%)	Overall (N = 249)	Group 1	Group 2	Group 3	*p*-value
		eGFR <30 mL/min/1.73 m^2^	eGFR 30–59 mL/min/1.73 m^2^	eGFR ≥60 mL/min/1.73 m^2^	
		(N = 70, 26%)	(N = 123, 46%)	(N = 56, 21%)	
All-cause Death	**57 (25)**	**23 (37.7)**	**27 (23.2)**	**7 (13.7)**	***p* = 0.012**
HF Hospitalization	67 (31.5)	18 (32.7)	34 (31.2)	15 (30.6)	*p* = 0.970
Death and HF Hospitalization	110 (48.2)	33 (54.1)	56 (48)	21 (42)	*p* = 0.444

eGFR, estimated glomerular filtration rate; HF, heart failure. Statistically significant variables have been highlighted in bold.

### 3.4 Univariable and Multivariable Analysis

As summarized in Table 5, in univariable analysis the eGFR is the only significant factor among the clinical variables considered. This data was also confirmed in multivariable analysis, despite showing only trends towards significance after adjusting also for sex and age. Of note, in the multivariable analysis TAPSE, left ventricle ejection fraction (LVEF), MR severity, history of atrial fibrillation/atrial flutter, age and sex were not significant factors.

**Table 5.** **Univariable and multivariable analysis**.

Variable		Univariate			Multivariable Model 1			Multivariable Model 2		
		HR	95% CI	*p*-value	HR	95% CI	*p*-value	HR	95% CI	*p*-value
eGFR										
	Moderate RD vs. Severe RD	**0.55**	**(0.31–0.96)**	**0.036**	**0.51**	**(0.26–0.96)**	**0.039**	0.57	(0.28–1.15)	0.116
	Mild RD vs. Severe RD	**0.33**	**(0.14–0.78)**	**0.012**	**0.25**	**(0.08–0.77)**	**0.016**	0.27	(0.07–1.02)	0.054
TAPSE		0.94	(0.87–1.02)	0.120	0.96	(0.88–1.04)	0.347	0.93	(0.84–1.02)	0.105
LVEF		0.98	(0.96–1.00)	0.186	1.00	(0.98–1.02)	0.674	0.99	(0.96–1.02)	0.367
MR Severity										
	Moderate-Severe (3+)	0.88	(0.20–3.75)	0.872	0.89	(0.20–3.94)	0.882	1.23	(0.26–5.89)	0.796
	Severe (4+)	0.44	(0.91–2.14)	0.311	0.52	(0.09–2.75)	0.445	0.64	(0.10–4.33)	0.651
Atrial Fibrillation/Atrial Flutter		1.08	(0.55–2.14)	0.814	1.34	(0.51–3.53)	0.542	2.01	(0.58–7.00)	0.274
Age		1.03	(0.99–1.07)	0.319	–	–	–	1.02	(0.97–1.07)	0.411
Male vs. Female		0.76	(0.44–1.30)	0.121	–	–	–	0.48	(0.21–1.08)	0.076

HR, hazard ratio; CI, confidence interval; eGFR, estimated glomerular filtration rate; RD, renal dysfunction; TAPSE, tricuspid annular plane systolic excursion; LVEF, left ventricle ejection fraction; MR, mitral regurgitation. Statistically significant variables have been highlighted in bold.

## 4. Discussion

This study shows that in patients with severe TR and chronic HF, severe RD (eGFR <30 mL/min/1.73 m^2^) is associated with increased all-cause mortality during a median follow-up period of 279 days, in comparison with patients with moderate or mild/no RD (Fig. 1, Log-rank *p* = 0.014) and this was independent of other risk factors.

Among non-cardiovascular comorbidities in HF, CKD plays an important role in term of incidence and prognosis [13, 14]. RD in HF with preserved ejection fraction (HFpEF) may be regarded as a major comorbidity, having a general prognostic impact independent of worsening HF status. In contrast, in patients with HF with a reduced ejection fraction (HFrEF), kidney dysfunction may indicate the progression of HF, likely due to factors such as low cardiac output, hemodynamic hypoperfusion, and activation of the sympathetic and neurohormonal systems. However, previous data suggested that RD posed a clinically significant risk for increased mortality in patients with HF [15]. CKD has been linked to worse outcomes across all HF phenotypes; however, studies on mortality in patients with HF with HFpEF and CKD have yielded mixed results. A large meta-analysis including a cohort of HFpEF patients found that CKD was a stronger predictor of death [16]. On the other hand, the Global Group in Chronic Heart Failure (MAGGIC) meta-analysis showed that patients with HFpEF had a lower mortality rate and a weaker association between CKD and death compared to those with HFrEF [17]. This finding was further supported by the Swedish Heart Failure registry, where the link between CKD and mortality risk was less pronounced in HFpEF patients [18]. However, in HF patients CKD was the disease more frequently associated with hospitalization and poor quality of life [19, 20].

In our study eGFR is an independent predictor of all-cause mortality in univariable and multivariable analysis, comparing patients with moderate RD vs. those with severe RD (HR 0.51; 95% C.I. 0.26–0.96; *p* = 0.039) and patients with mild RD vs. those with severe RD (HR 0.25; 95% C.I. 0.08–0.77; *p* = 0.016), despite showing only trends towards significance after adjusting also for sex and age.

The results of our study reflect the literature data regarding the all-cause mortality in HF patients, independently of ejection fraction. In fact, despite the ejection fraction significantly differing between the 3 groups of patients stratified for eGFR with lowest LVEF in patients with severe RD (41.7 ± 13% vs. 45.0 ± 14% vs. 48.5 ± 13.1%, *p* = 0.021), in the multivariate analysis this parameter was not a significantly negative prognostic factor (HR 1.00; 95% C.I. 0.98–1.02; *p* = 0.674). Similarly, TAPSE was significantly lower in patients with severe RD (16.7 ± 4 mm vs. 17.6 ± 4.0 mm vs. 19.8 ± 4 mm, *p* < 0.001) but this parameter did not appear a predictive factor in both univariable (HR 0.94; 95% C.I. 0.87–1.02; *p* = 0.120) and multivariable analysis (HR 0.96; 95% C.I. 0.88–1.04; *p* = 0.347). However, the effect of additional confounding factors remains to be established.

No significant difference was observed in HF hospitalizations between the 3 groups of patients (32.7% vs. 31.2% vs. 30.6%, *p* = 0.970) and the incidence of the composite outcome of all-cause mortality or HF hospitalization was similar when comparing groups (54.1% vs. 47.9% vs. 42.0%, *p* = 0.444). The latter result seems to be mostly driven by HF hospitalization and this finding could be affected by several confounders. Furthermore, at Kaplan-Meier analysis the composite endpoint of all-cause mortality or HF hospitalization tended to be higher in patients with worse RD (log-rank *p* = 0.073) while no difference was found for HF hospitalization alone (log-rank *p* = 0.916) (Fig. 2A,B).

Looking at the baseline characteristics of the overall population, it is evident that we are dealing with a very elderly population (mean age: 79.3 ± 9.0 years) with a high burden of comorbidities.

This data could have an important impact on HF hospitalizations in such a way as to show no differences in the population stratified by eGFR. Moreover, the high burden of comorbidities that guide the prognosis of these patients could explain why some prognostic factors such as LVEF, TAPSE, severity of MR and atrial fibrillation (AF)/atrial flutter (AFL) were not significant in both univariate and multivariate analysis of our study (Table 5).

In relation to medical therapies (Table 3), the percentage of RAAS inhibitors drugs was very low in the overall population, especially in patients with severe RD (i.e., ACEi: 11.1% vs. 27.6% vs. 29.5%, *p* = 0.014). This was an expected finding given the preponderance of patients with moderate or severe RD. Moreover, in this type of patient, symptomatic hypotension represents an important clinical problem and often the use of RAAS inhibitors becomes prohibitive. On the other hand, we recorded a high percentage of beta-blocker use in the overall population (81.3%) without significant differences between the 3 groups of patients.

A remarkable difference was reported for the average dosage of loop diuretics, which was significantly higher in patients with severe RD (215.8 ± 33 mg vs. 149.1 ± 17 mg vs. 94.4 ± 13 mg, *p* = 0.013). This result is expected given the high prevalence of the diuretic resistance phenomenon in patients with advanced CKD and HF [21].

Interesting pathophysiological mechanisms mentioned earlier, linkedsystemic venous congestion to RD. Often, the fluid overload is due to right ventricular dysfunction which may be associated with primary or secondary TR.

TR is a very common valvular heart disease in chronic HF, with a global prevalence of about 19% in these patients [22, 23]. Furthermore, literature data reported that TR contributes to RD in patients with HF [8]. Irrespective of left ventricular function and pulmonary hypertension, TR is associated with increased morbidity and mortality, partly due to the development of right HF [24, 25, 26].

So, we focused our attention on a selected population of patients affected by severe TR and chronic HF. In our overall population, many patients had a secondary (or functional) TR (81.9%); only a minority of patients had a primary TR (7.3%) or Cardiovascular Implantable Electronic Device (CIED)-related TR (10.8%).

In recent years, TR in HF patients has gained much attention from the scientific community, above all thanks to the strong interest of device manufacturers towards development of transcatheter therapies, which may offer a safe and effective alternative to surgery in this high-risk population [27, 28].

In recent years, several minimally invasive transcatheter-based techniques have been developed to reduce TR [29, 30, 31, 32]. Although initially promising, most transcatheter tricuspid valve replacement (TTVR) methods are still in the developmental stage, and comprehensive acute or long-term follow-up data are limited [33]. Currently, the most commonly used technique is transcatheter edge-to-edge repair (TEER) of the tricuspid valve [34]. Retrospective analyses indicate that this approach effectively reduces TR and alleviates symptoms [35, 36]. The Trial to Evaluate Cardiovascular Outcomes in Patients Treated with the Tricuspid Valve Repair System Pivotal (TRILUMINATE Pivotal) evaluated the safety and performance of a TEER system (TriClip [Abbott, Chicago, IL, USA]), for the treatment of patients with symptomatic moderate or greater TR who were deemed to be at high risk for tricuspid valve surgery with valve anatomies that were considered appropriate for transcatheter edge-to-edge repair. The trial successfully met both primary safety (composite of major adverse events at 6 months) and performance (TR reduction at 30 days) endpoints, which were previously reported [27]. The repair proved durable in reducing TR at 1 year and was associated with sustained and significant clinical benefits, including low mortality after 1 year, in a high-risk, vulnerable population [37].

However, not all patients with significant TR have favorable valve anatomy to be treated with a TEER system. For this reason, other transcatheter therapeutic options have been validated. Of note, heterotopic bicaval stenting is an emerging, attractive transcatheter solution for these patients. In the TRICUS EURO (Safety and Efficacy of the TricValve® Transcatheter Bicaval Valves System in the Superior and Inferior Vena Cava in Patients With Severe Tricuspid Regurgitation) trial, the dedicated bicaval system for treating severe symptomatic TR was associated with a high procedural success rate and significant improvements in both quality of life (QOL) and functional classification at 6 months follow-up [38].

Unfortunately, in the diagnostic work-up of these non-TEER procedures, CT scan is often mandatory but remains prohibitive in patients with severe RD. In our study population, about 30% (patients with severe RD) could not benefit from these treatments.

In this context the RD, in a selected population with severe TR and chronic HF, plays an additional negative prognostic role [9]. Therefore, delivering a similar treatment to these patients could be futile and should be managed conservatively. Indeed, the pharmacological therapy for heart failure available to date has demonstrated benefits, especially in patients with HFrEF [39].

Potential limitations should be considered in the interpretation of the discussed data. This study was retrospectively conducted in two high volume centers, and thereby may be subject to selection bias. Given the observational design and the low number of patients included, which limits the power of some analysis, causality should not be inferred from our data. All-cause mortality was chosen as the primary endpoint due to the lack of systematic recording of the exact cause of death. Nevertheless, we applied stringent inclusion criteria to enhance the validity of our model. Patients were enrolled after an initial echocardiographic and clinical assessment, so some may have had functional TR prior to enrollment. This limitation is common in studies on this clinical field. Additionally, a single echocardiographic measurement of TR severity at rest may not fully capture the true severity of functional TR, which can vary with changes in right ventricular preload, afterload, and contractility. Accurately assessing right ventricular dysfunction in the context of significant TR is complex, as TR-induced right ventricular unloading can be misleading. Furthermore, relying on a single echocardiographic parameter such as TAPSE to evaluate right ventricular function can be problematic, as TR may impact the systolic excursion of the tricuspid annular plane [40].

Another potential limitation of our study is that in the definition of RD, we did not include albuminuria values ​​because they were not available. Patients were therefore classified only based on their eGFR value and this could cause an underestimation of patients with RD.

Finally, there are missing data among the 3 groups and the follow-up period is relatively short and incomplete. There was not a clinical adjudication committee.

## 5. Conclusions

Severe RD is present in about 30% of a contemporary cohort of patients with severe TR and chronic HF and is associated with increased incidence of all-cause mortality at mid-term follow-up, when compared with moderate and mild/no RD, regardless of RV function. No significant difference in HF hospitalizations alone or in the composite endpoint including also all-cause mortality was evident between groups. Prospective studies assessing the impact of RD in patients with TR and chronic HF are needed to confirm our hypothesis and to better identify patients that could benefit from transcatheter or surgical therapies.

## Data Availability

The datasets used and/or analyzed during the current study are available from the corresponding author on reasonable request.

## Abbreviations

RD, renal dysfunction; HF, heart failure; HFpEF, heart failure with preserved ejection fraction; HFrEF, heart failure with reduced ejection fraction; FTR, functional tricuspid regurgitation; TAPSE, tricuspid annular plane systolic excursion; NYHA, New York Heart Association; CKD-EPI, Chronic Kidney Disease Epidemiology Collaboration; BMI, body mass index; AMI, acute myocardial infarction; PCI, percutaneous coronary intervention; CABG, coronary-artery by-pass graft; TIA, transient ischemic attack; COPD, chronic obstructive pulmonary disease; AF, atrial fibrillation; AFL, atrial flutter, BNP, brain natriuretic peptide; NT-proBNP, N-terminal pro brain natriuretic peptide; STS, Society of Thoracic Surgery; ICD, implantable cardioverter defibrillator; CRT, cardiac resynchronization therapy; LA, left atrial; LVEDD, left ventricle end-diastolic diameter; LVEDV, left ventricle end-diastolic volume; LVEDVi, indexed left ventricle end-diastolic volume; LVEF, left ventricle ejection fraction; MR, mitral regurgitation; TR, tricuspid regurgitation; CIED, Cardiovascular Implantable Electronic Device; RVEDD, right ventricle end-diastolic diameter; RVEDV, right ventricle end-diastolic volume; RVEF, right ventricle ejection fraction; RA, right atrium; sPAP, systolic pulmonary arterial pressure; RAAS, renin-angiotensin-aldosterone system; ACEi, angiotensin-converting enzyme inhibitors; ARBs, angiotensin receptor blockers; ARNI, angiotensin receptor neprilysin inhibitor; MRAs, mineralocorticoid receptor antagonists; SGLT2i, Sodium-glucose co-transporter-2 inhibitors; HR, hazard ratio; CI, confidence interval; eGFR, estimated glomerular filtration rate; TAPSE, tricuspid annular plane systolic excursion; LVEF, left ventricle ejection fraction; MR, mitral regurgitation; MAGGIC, meta-analysis of the Global Group in Chronic Heart Failure; TTVR, trans-catheter tricuspid valve repair; TRILUMINATE Pivotal, Trial to Evaluate Cardiovascular Outcomes in Patients Treated with the Tricuspid Valve Repair System Pivotal; TEER, transcatheter edge-to-edge repair; TRICUS EURO, Safety and Efficacy of the TricValve® Transcatheter Bicaval Valves System in the Superior and Inferior Vena Cava in Patients With Severe Tricuspid Regurgitation; QOL, quality of life.

## Supplementary Material

In the supplementary materials section (online available), the technical specifications used for echocardiographic measurements and the method used to estimate the severity of valvular diseases are indicated. Furthermore, Kaplan – Meier curves for the composite endpoint (all-cause mortality and HF hospitalization) and Kaplan – Meier curves for first HF hospitalization are reported in this section. Supplementary material associated with this article can be found, in the online version, at https://doi.org/10.31083/RCM26080.
