# Supplementary material for: Clinical Impact of Renal Dysfunction in Patients with Severe Tricuspid Regurgitation and Chronic Heart Failure
Source: Rev Cardiovasc Med. 2025 Mar 5;26(3):26080. doi: 10.31083/RCM26080 (PMC11951283; doi:10.31083/RCM26080)
Supplement: Supplementary file 1 [file 2153-8174-26-3-26080-s1.docx]

**Supplementary Materials**

**Echocardiographic study**

The severity of mitral regurgitation was graded with quantitative measurements using at least one of the following methods: proximal isovelocity surface area (PISA) or vena contracta width. Mitral regurgitation was classified as mild [effective regurgitant orifice area (EROA) <0.20 cm^2^, vena contracta width <0.3 cm], moderate (EROA ≥0.20 or <0.39 cm^2^, vena contracta width ≥0.3 or <0.69cm), or severe (EROA ≥0.40cm2, vena contracta width ≥0.7 cm) [28]. When the two methods were discordant in defining the degree of mitral regurgitation severity, the value of EROA was accepted as the reference value. Left ventricular dimensions were evaluated with left ventricular end-diastolic and end-systolic volumes, whereas left ventricular function with ejection fraction was calculated with the biplane Simpson’s method. Diastolic function was evaluated by transmitral Doppler. Systolic pulmonary arterial pressure (sPAP) was derived from the peak velocity of the tricuspid regurgitation jet and adding the estimation of right atrial pressure (RAP). TR was quantified by an integrated approach. Echocardiographic parameters used for grading included the following: vena contracta width (mild <0.3cm, moderate ≥0.3 or <0.69 cm, or severe ≥0.7 cm); area/right atrial area ratio tricuspid regurgitation (mild <19%, moderate ≥20 or <40%, or severe ≥40%); hepatic venous flow pattern; and right atrial size [28]. Right ventricular dimensions were evaluated with the end-diastolic mid-ventricular diameter. Right ventricular systolic function was assessed with TAPSE, and a value less than 16 mm was accepted as indicative of right ventricular systolic dysfunction. RAP was estimated by inferior vena cava (IVC) diameter and the presence of inspiratory collapse as follows [30]: IVC diameter 2.1 cm or less that collapses more than 50% with a sniff suggests a normal RAP (range 0 – 5 mmHg), whereas an IVC diameter greater than 2.1cm that collapses less than 50% with a sniff suggests a high RAP (range 10 – 20 mmHg); in indeterminate cases in which the IVC diameter and collapse do not fit this paradigm, an intermediate value of RAP (range 5–10mm Hg) was used.
